# The impact of the COVID-19 pandemic on the mental health of new mothers in China: A qualitative study of mothers with infants aged 0–1 year old

**DOI:** 10.3389/fpubh.2023.1138349

**Published:** 2023-03-27

**Authors:** Dandan Zou, Chen Chen

**Affiliations:** ^1^School of Marxism, Xi'an University of Technology, Xi'an, China; ^2^College of Literature and Journalism, Qiannan University of Science and Technology, Qiannan, China

**Keywords:** COVID-19, mother-infant interactions, mental health, anxiety, public health

## Abstract

**Background:**

China has implemented a strict epidemic control policy (ECP) for 3 years during the COVID-19 pandemic. New mothers are under great psychological pressure to protect themselves against the virus, following the ECP, as well as taking on the main responsibility of raising their children. However, the mental health of this group has been neglected by the public. This article aims to understand the mental health of new mothers during the COVID-19 pandemic.

**Method:**

Qualitative research methods were adopted in this study. From 1 October to 1 November 2022, we conducted in-depth interviews with 36 new mothers in Guiyang, Guizhou, China, and used thematic analysis to examine their emotional status, as well as the origins of their negative and positive emotions.

**Results:**

(1) New mothers are chronically depressed, feeling anxious, and upset. (2) Negative emotions are caused either by the virus or by the ECP. (3) New mothers are mainly anxious about their children's physical health, feeding options, childcare, and family income. (4) Positive emotions are reflected by tight parent–child bonds, a better understanding of childcare, and an increased ability to perceive risks.

**Conclusion:**

The anxiety of new mothers has revealed the shortcomings of the Chinese health system in the emergency management of the mother and child. At the same time, the outbreak is an opportunity to improve the response management capacity of the health system in order to prevent the recurrence of similar problems for mothers and infants.

## 1. Introduction

The World Health Organization (WTO) declared COVID-19 a global pandemic on 11 March 2020 (1). As the virus has mutated, it has become less lethal but more infectious, and the majority of patients are able to recover by themselves (2). It is clear that the virulence of COVID-19 has been dismissed and is continuously dismissing further (3). Nevertheless, the economic and health impacts caused by COVID-19 are ongoing (4). It is likely there will be significant and extensive social change in the coming years (5). During the pandemic, most countries implemented a short-lived control policy based on the concept of living with the virus. Nevertheless, China has been implementing a strict ECP since the outbreak of COVID-19 in December 2019. In other words, if there is a positive or suspected case, the person or community with whom the patient is in contact has to be isolated or shut down. As a result, following this strict ECP for 3 years, many families have experienced psychological changes due to the severe impact on their living conditions (6). Mothers of newborns during the epidemic were faced with the dual challenges of ECP and childcare, which brought them great psychological stress. COVID-19 is not the first virus to threaten humans, nor will it be the last (7). Therefore, it is essential to understand the mental health of new mothers during the epidemic. (1) This facilitates the identification of psychological problems arising from deficiencies in ECP, promotes the humanization of policies, and improves the capacity to cope with the public crisis. (2) It is beneficial for the government to promote a better healthcare system that takes full account of the rights of infants and creates a healthy social environment. (3) It helps to develop women's mental health and provides psychological guidance on time. (4) It is conducive to forecasting the rate of fertility, stimulating the government to adjust the policy and reduce the costs of fertility.

There is no doubt that the COVID-19 pandemic has caused much emotional stress to many families (8). Postnatal mothers may have suffered some mental distress during the epidemic (9, 10). In the study by Kochan et al. all postnatal women who gave birth during that period were reported to have been negatively affected by COVID-19 (11), which is reflected in maternal adjustment, maternal attachment, and baby care after childbirth (12). There are two main causes of this negative emotion, one being anxiety about the infant's infection. A study showed that there were statistically significant differences between fear about being infected with COVID-19 for themselves and babies and postpartum depression (13). In a retrospective analysis of infected mothers, it was found that only a small proportion of newborns were confirmed positive (14), that is, close contact between mother and infant can transmit the virus to the infant through droplets or microdroplets (15). However, it has also been suggested that the effect of an infected mother on her newborn infant is unknown (16), and there is no evidence of vertical transmission of COVID-19 (17, 18). This is because there are limited published data on infants infected in the neonatal period and the long-term effects of the virus on newborns are still unknown (19). Another is the fear of the clinical manifestations of the infection. Many studies have found that most newborns infected after birth have gastrointestinal or respiratory symptoms (20). Yaman et al. observed some infected newborns and found that the most common symptoms were feeding intolerance, cough, elevated fever, and respiratory distress (21). It may also occur as a rare case of multisystem inflammatory syndrome in children in newborns due to COVID-19—presenting with stage 11 b necrotizing enterocolitis (22). Any symptom can cause anxiety in mothers.

More stress is associated with parenting during the parenting period. Parents often feel lonely and isolated with a decreased sense of wellbeing (23). Some families also reported an increase in depression (24). This is due to prolonged lockdown and social isolation which exacerbates parenting burnout (25). For example, parents were socially isolated during lockdown (7) and, thus, did not receive professional and experienced parenting guidance on time (26), which has caused changes in family behaviors (27). A study also stated that social isolation leaves infants with potential deficits in social communication skills (28). Okinarum and Rochdia noted that the weakened breastfeeding experience during the COVID-19 pandemic was dominated by impaired comfort, inadequate milk supply, parenting problems, and indifferent spouses (29). The most vulnerable mothers of the future are those whose income has been reduced as a result of the COVID-19 pandemic (30).

Women in pregnancy also face significant mental health challenges (31) and high levels of stress and anxiety throughout pregnancy (32). Their fragile psychology undermines the formation of a positive relationship with their unborn babies (33), the COVID-19 has affected their ability to purchase items for their babies and their feeding schedule (34).

The positive and negative emotions of new mothers in raising their children during the COVID-19 pandemic were explored in this study under the combination of the existing research results and the context of China's special ECP.

## 2. Materials and methods

### 2.1. Study design

This study uses qualitative methods to understand the mental health of new mothers during the COVID-19 pandemic. Qualitative research is considered to explain a specific problem by uncovering issues, understanding the phenomenon of events, and analyzing human behaviors (35, 36). It can focus on how an individual experiences a specific phenomenon (37, 38). Our study selected new mothers in Guiyang, Guizhou, China. On 2 September 2022, there was a confirmed case of COVID-19 in Guiyang, and on 5 September, the entire city started the ECP which was reopened on 19 September. Therefore, we decided to interview and investigate the group of new mothers who have experienced this special period, to identify their actual feelings about raising children.

### 2.2. Data collection procedure

(1) The author searched for 10 maternity centers in Guiyang (a maternity center is a place that provides professional postnatal recovery services for mothers who have given birth, and is traditionally used in many Asian countries, such as China, Japan, Korea, Singapore, etc.), explained the intention to the managers, and finally found two centers that agreed to participate in the interview. (2) On the recommendation of the manager, the author joined the WeChat group of mothers who have returned home from their monthly stay in the center. The group includes women who gave birth between 2020 and 2022, which is appropriate for this study. Volunteers were recruited from the group and 36 new mothers were found eventually. (3) Interview purpose: to examine the impact of the COVID-19 pandemic on the mental health of new mothers. (4) Interview duration: 1 October to 1 November 2022. (5) Interview process: we conducted in-depth interviews (semi-structured interviews) with 36 new mothers through voice chat on WeChat around the interview questions. Each interview lasted ~1 h and the language used in each interview was Chinese. Each interview was recorded with the consent of each interviewee.

### 2.3. Data analysis

(1) Data cleaning: after the interviews, the recordings were transcribed into texts in Chinese. Then, the data were preliminarily cleaned by deleting the blanks, unrecognized words, incorrect words, and unclear expressions appearing in each conversion. Finally, a set of interview data featured with smooth Chinese wordings was obtained and translated into English. (2) Code generating: we reviewed the data repeatedly to increase our familiarity. Then, the interview data was put into NVIVO 12 software for coding. The name of each interviewee was anonymized. While the data were being encoded, our team often held online meetings to discuss themes and our unique findings using inductive methods. When all the data were encoded, our coding work was completed. (3) Review the themes: the researchers reviewed the coding of each theme to verify whether they formed a coherent pattern of research questions to ensure that the logic was clear and whether they described the meaning of the entire dataset. (4) Conducting the thematic analysis: in this step, the authors identified the final themes and subthemes and wrote the report explaining how the findings were developed as well as presenting a final interpretation of the theme naming and interview data. (5) Credibility: first, there was a peer debriefing with three university professors who specialize in postnatal psychology. As a result, they examined all processes of data collection, collation, and thematic analysis. In addition, it has been examined through member checks to accurately examine the interpretations and themes that emerged from the interviews, with quotations from interviewees provided in the findings to enhance credibility. Finally, it has adopted the triangulation techniques to decrease the impact of potential bias in the data.

## 3. Results

**Figure 1:**
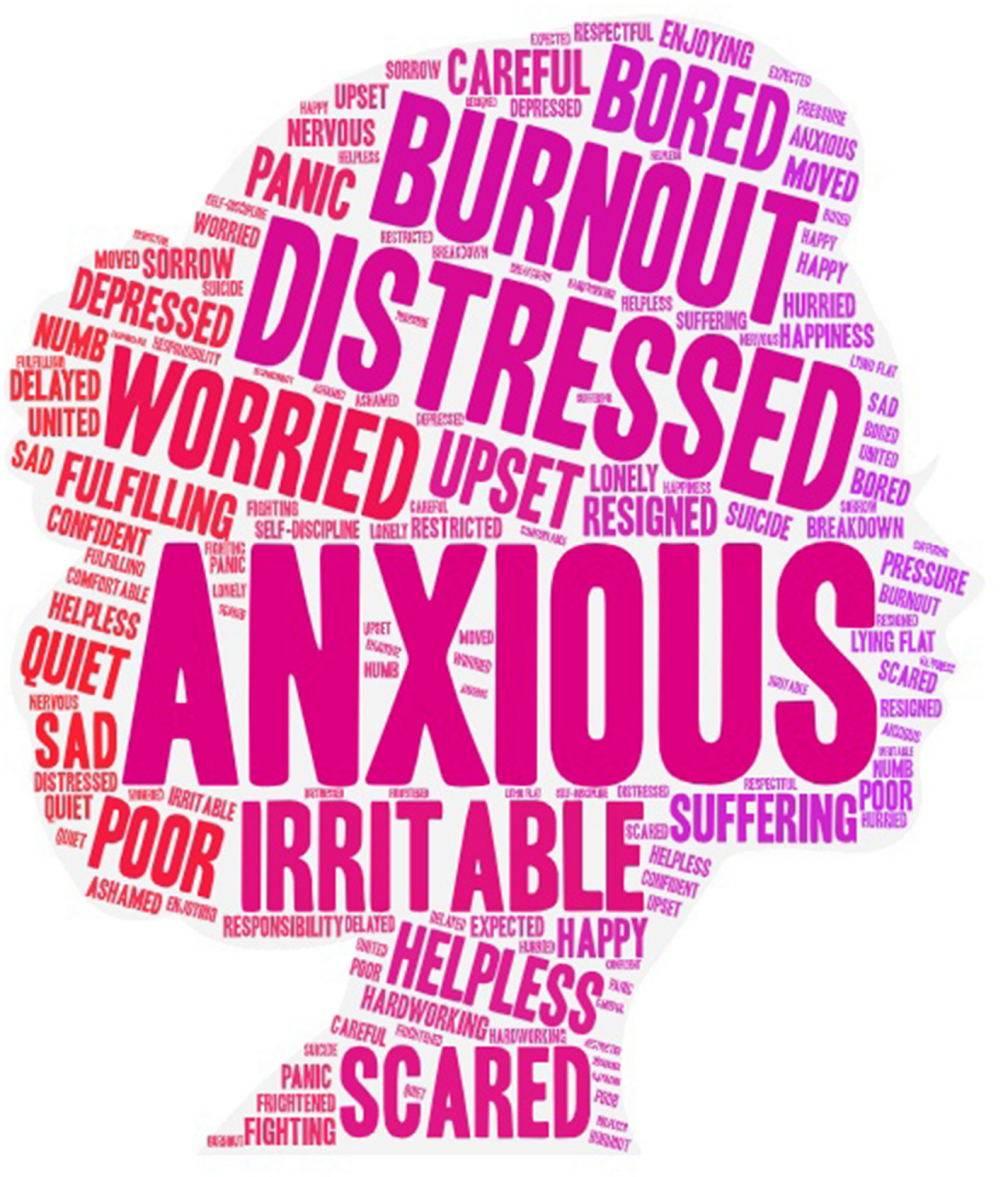
The emotional word cloud.

Table 1 shows the sociodemographic characteristics of the qualitative study participants (*n* = 36). Of these, 75% of the new mothers were vaccinated, and 100% of them had a bachelor's degree or even higher, with a high level of educational background that indicates a good understanding of the interview questions and provides in-depth insights. Figure 1 shows a graph of the emotional word cloud for parenting during the pandemic for new mothers, where 81% are negative emotions and 19% are positive emotions. The top three negative emotion words were anxious, distressed, and burnout. The top three positive emotion words were fulfilling, happy, and hardworking. Table 2 shows the graph of the analysis results, i.e., themes and sub-themes.

**Table 1:** Sociodemographic characteristics of the qualitative study participants (*n* = 36).

**Code**	**Age**	**Education background**	**Vaccination**	**Age of child in months (as of 1st October 2022)**	**Number of children**	**Description of the emotional word**
A1	26	Bachelor's degree	Yes	2	1	Breakdown, enjoying, fulfilling
A2	33	Bachelor's degree	No	6	2	Anxious, responsibility, happiness
A3	28	Bachelor's degree	No	3	1	Burnout, irritable, scared
A4	30	Master's degree	Yes	5	1	Depressed, bored, worried
A5	28	Bachelor's degree	Yes	6	2	Depressed, suffering, irritable
A6	31	Bachelor's degree	No	3	1	Panic, scared, irritable
A7	25	Bachelor's degree	Yes	4	1	Panic, scared, worried
A8	31	Bachelor's degree	Yes	5	2	Panic, worried, sad
A9	26	Bachelor's degree	No	2	1	Bored, worried, anxious
A10	34	Master's degree	Yes	4	2	Anxious, careful, quiet
A11	26	Bachelor's degree	Yes	4	1	Distressed, anxious, self-discipline
A12	30	Bachelor's degree	Yes	6	1	Careful, anxious, helpless
A13	31	Bachelor's degree	Yes	8	1	Restricted, bored, worried
A14	27	Bachelor's degree	Yes	7	2	Resigned, distressed, delayed
A15	26	Bachelor's degree	No	3	1	Depressed, resigned, pressure
A16	29	Master's degree	Yes	4	1	Lying flat, moved, numb
A17	28	Bachelor's degree	Yes	11	1	Anxious, helpless, expected
A18	25	Bachelor's degree	Yes	7	1	Anxious, irritable, suffering
A19	31	Bachelor's degree	Yes	6	1	Suicide, ashamed, helpless
A20	27	Bachelor's degree	Yes	5	1	Scared, hurried, worried
A21	30	Bachelor's degree	No	3	3	Happy, quiet, fulfilling
A22	27	Master's degree	Yes	10	1	Happy, comfortable, fulfilling
A23	26	Bachelor's degree	Yes	2	1	Hardworking, respectful, united
A24	26	Bachelor's degree	Yes	5	2	Suffering, distressed, sorrow
A25	28	Bachelor's degree	No	3	1	Frightened, anxious, helpless
A26	27	Bachelor's degree	Yes	6	1	Worried, hardworking, distressed
A27	28	Bachelor's degree	Yes	12	1	Burnout, fighting, confident
A28	33	Bachelor's degree	Yes	5	2	Bored, upset, distressed
A29	31	Bachelor's degree	Yes	7	1	Poor, nervous, irritable
A30	30	Bachelor's degree	Yes	8	1	Distressed, burnout, irritable
A31	28	Master's degree	Yes	10	1	Irritable, confident, burnout
A32	27	Bachelor's degree	No	3	1	Distressed, lonely, poor
A33	30	Bachelor's degree	Yes	10	2	Burnout, upset, sorrow
A34	30	Bachelor's degree	Yes	7	1	Helpless, poor, nervous
A35	28	Bachelor's degree	Yes	8	3	Fighting, poor, burnout
A36	26	Master's degree	No	5	1	Distressed, burnout, upset

**Table 2:** Themes and sub-themes.

**Themes**	**Sub-themes**
Health	Being infected
	Being isolated
	Outbreak of other diseases
	Delayed plans
Feeding problems	Nutrition in breast milk
	Shortage of formula milk
	Baby supplies
	Types of infant foods
Parenting burnout	Lack of toys
	Lack of sleep
	Sports and socializing
Family income	Declining family income
	Increasing family costs
Family time	Parent-child companionship
	Parent-child interaction
Health knowledge	COVID-19 vaccine
	Risk perception

### 3.1. Health

#### 3.1.1. Being infected

The greatest anxiety among the interviewees was about being infected, as they were concerned about three main subjects: their children, themselves, and strangers. The psychological burden is exacerbated by the symptoms of the child's infection and the uncertainty of the after-effects.

“*Fevers are difficult for infants to tolerate, as well as the treatment process, medication for small infants may cause damage to their liver with its after-effects” (A2)*.

“*I'm afraid I'll get infected and pass it on to my children” (A3)*.

Strangers were one of the risk factors that potentially influenced the choice of a nanny by the interviewees. They indicated that they would feel at ease if they had a nanny, especially women in the postnatal period.

“*With so many people infected, I struggle to find good nannies to look after the children for me and share the household chores” (A1)*.

#### 3.1.2. Being isolated

The interviewees were very frustrated by the isolation and the uncertainty of the isolated places, which might be a five-star hotel or a school dormitory in the village. Generally speaking, isolation is required urgently by the government, as to whether sufficient baby supplies can be provided at the quarantine places, as well as whether the babies will be able to adapt to the unfamiliar environment and whether there is a risk of being infected by the gathering during the transfer. Interviewees stated that everything was uncertain.

“*Once there was a confirmed or suspected case nearby, we would be forcibly pulled to a designated place for quarantine. Whilst my child is still an infant, the quarantine place would be lack of convenient baby supplies, which makes it difficult to stay” (A6)*.

“*Instead, the quarantine area increases the risk of being infected. Thus, all dare not catch a cold now” (A8)*.

#### 3.1.3. Outbreak of other diseases

The interviewees felt that the nucleic acid testing policy was dehumanizing and overly rigid. The reason is that a 24-h nucleic acid testing is now required to enter the hospital. This means that both the parents and the child must have their nucleic acid testing done in advance and then wait for the nucleic acid results to show negative. The problem is that it is unpredictable for infants to have emergencies. In addition, it usually takes about 8 h for the nucleic acid results to be back, which takes too long for an infant with a sudden disease.

“*If there is an emergency like diarrhea or high fever. I am afraid that my child will not get timely treatment if the nuclear results come out too slowly. Especially at night when the nucleic acid tests take longer, and the medical staff are understaffed” (A11)*.

#### 3.1.4. Delayed plans

Travel restrictions during the ECP disrupted the parenting plans of the interviewees, such as the physical examination and child vaccinations. Infants who are growing too fast are bound to have inaccurate data from overtime physical examinations. Moreover, some vaccines have limited injection times.

“*The physical examination was delayed and may not have detected physical problems in time or the need for nutrition” (A15)*.

“*We have missed the best time to get the children's vaccinations” (A23)*.

### 3.2. Feeding problems

During the ECP, there was a severe shortage of household supplies due to the suspension of logistics and urban deliveries. This included adult supplies, baby formula milk, and baby supplements. However, the need for these had to be requested from the community manager, which made it difficult to get what was once readily available, and the interviewees found it difficult to have balanced nutrition.

#### 3.2.1. Nutrition in breast milk

Official regulations stipulate that household goods can only be purchased online at government-designated supermarkets, and the suspension of logistics and urban deliveries has caused a severe shortage of supplies.

“*Now it's not about buying whatever you want, you can only buy what is available. If I don't eat enough and my breast milk will not be nutritious” (A4)*.

#### 3.2.2. Shortage of formula milk

Because of the ECP, all shops in the city had to be closed as well as logistics and deliveries were both stopped, thus, there was a lack of staff to deliver formula milk even if it was available.

“*It is very troublesome to apply to the community managers to buy formula milk” (A36)*.

#### 3.2.3. Baby supplies

There is a severe shortage of baby supplies and a lack of staff to deliver them if they are available.

“*It is difficult to purchase baby supplies such as diapers, clothes, milk bottles, etc” (A25)*.

#### 3.2.4. Types of infant foods

There was a lack of stock of foods for infants and the tools needed to make infant foods during the ECP. Interviewees indicated that they could only add supplementary foods later for infants who were ready to have them.

“*It is difficult to buy the vegetables and meat to make a varied meal that you want, and the diet structure is not balanced. The time of the ECP is unclear, which is likely to be around two months” (A18)*.

### 3.3. Parenting burnout

#### 3.3.1. Lack of toys

All interviewees felt a strong sense of parenting burnout, lacking the assistance of nannies and toys so that they could only concentrate on playing with the children. Especially with two children at home, taking care of both the older and the younger one certainly increases the mental burden on parents.

“*We can't buy new toys to relieve the stress of being with children” (A12)*.

“*I am very tired of taking care of two children at the same time. During the ECP, neither could I go out nor buy toys. I felt helpless and devastated” (A10)*.

#### 3.3.2. Lack of sleep

Many of the interviewees were in short of sleep and depressed. Although it was common for infants aged 0–1 year to wake up several times, both night awakening and nursing affected the quality of sleep of the interviewees. During non-epidemic periods, mothers were able to take their children out for a relaxing walk, which helped the infants to sleep. However, during the ECP, it was a lot more tiring for mothers to have to spend time with their children from morning to night.

“*During the ECP, the universal nucleic acid testing policy was implemented, and two tests were required for 3 days. In order to ensure that all residents got their nucleic acid results on the same day and faced with a large population base, we were asked to do the test at 5 a.m. every day, which seriously affected the quality of sleep of our children” (A16)*.

“*I was unable to walk my child with the pram, whereas going out and being in touch with new things helped him to sleep. Outdoors is also a time for me to relax, unluckily now I have to stay at home” (A5)*.

#### 3.3.3. Sports and socializing

During the pandemic, the government called for fewer gatherings, so many people spontaneously cut down on outdoor activities. Travel was even restricted in the ECP.

“*It is the time for children to practice walking and to play with other children to develop social skills, however, outdoor activities are reduced because of the fear of being infected” (A22)*.

### 3.4. Family income

#### 3.4.1. Declining family income

It has been found that the interviewees' income has declined but they are unable to do anything about it, as they are struggling to take care of their children and work at the same time.

“*My family income has seriously declined” (A9)*.

#### 3.4.2. Increasing family costs

During the ECP, high costs of logistics and deliveries led to a rise in the price of products, which consequently increased the cost of the family.

“*Buying formula milk, diapers and toys during this time is much more expensive than before” (A13)*.

### 3.5. Family time

Interviewees indicated that the only relief during the epidemic or ECP was the extra family time, which allowed parents to see the growth of their children.

#### 3.5.1. Parent-child companionship

“*I went out less, and with the restrictions on travel during the ECP, I had more time to spend with my children” (A33)*.

#### 3.5.2. Parent-child interaction

“*I have more time to train my child to lift his head, roll over, crawl, walk and talk” (A20)*.

### 3.6. Health knowledge

#### 3.6.1. COVID-19 vaccine

The interviewees acquired not only information about children's vaccines but also about the COVID-19 vaccine.

“*I have known about the COVID-19 vaccine and the importance of the vaccines for children” (A17)*.

#### 3.6.2. Risk perception

The interviewees were forced to learn about the protection of infectious diseases to ensure the safety of their infants. These 3 years also led them to promote their risk perception and highlighted the need for exercises.

“*We have purchased masks, protective clothing, and disinfectant medication, as well as we pay more attention to personal and children's sanitary problems. Children masks are also prepared when going out” (A21)*.

“*We have to prepare in advance for the various diseases that our children may face in the future” (A32)*.

“*I will put more emphasis on my child's exercise condition so as to strengthen the immune system” (A34)*.

## 4. Discussions

Our study describes the child-raising styles followed by new mothers in China during the COVID-19 pandemic. It was found that the COVID-19 pandemic has had a severe impact on the mental health of new mothers. Their negative emotions rose during the pandemic, which is consistent with the findings of previous studies. Different from previous studies, this study is novel and exploratory for several reasons. First, as far as we know, there is still no in-depth discussion on the mental health problems of new mothers in China during the COVID-19 pandemic. Previous studies have focused on children over 1 year old in China, mainly examining their vaccine (39), communication (40), sleep (41), and sports problems (42). In contrast, our research results supplement the feeding of infants aged 0–1 year in the context of the COVID-19 pandemic. Based on open-ended questions, we then summarized the sub-themes of each theme. The latest research results fully show the causes of anxiety in new mothers. Therefore, this study fills the gap in the existing articles on how the COVID-19 pandemic has led to difficulties for new mothers to raise young children. Second, our research results play a forward-looking role in predicting the future quantity and quality of the population, which has not been mentioned in previous studies. The number of births in China in 2020 is 18% lower than in 2019, and the total fertility rate falls to 1.3 (43). The changes in the number of births directly affected by the pandemic are concentrated in late 2020 and early 2021. Initially, the decline in fertility caused by the COVID-19 pandemic is the result of a combination of anxiety about the poor survival of infants, parenting burnout, a severe drop in household income due to the recession, the shutdown of work and production, and uncertainty and weakened confidence in future life expectations. These psychological problems directly affect their fertility intentions (44–46). It has been suggested that women who are chronically tired, frightened, depressed, and anxious may have weaker bonding with their families which may lead to more marital problems and mental disorders (47, 48). Therefore, it is important to be alert to the consequences of a population that remains at low fertility for a long time and intervenes in advance. The willingness to have children requires the capacity, measures, and effectiveness of the government's response to the pandemic, with regard to the state of economic recovery and growth, the speed and extent of people's productive recovery, and preferential policies to reduce the costs of childbirth, parenting, and education, as well as a well-developed health system. Interviewees expressed the expectation that the government would offer children a secure social environment in the future.

This study summarized the factors affecting the mental health of new mothers in China according to the interview results. In general, interviewees were anxious about the contagiousness of COVID-19, as well as being psychologically stressed by the strict ECP that affected their normal living conditions. This is because the policy is becoming increasingly demanding and appears to conflict with the interests of citizens (49–51). The mechanics of the policy are that, first, all febrile patients are classified as suspected, which leads to difficulties in getting medical treatment for common illnesses. Medical institutions should set green channels for the vulnerable group of infants and give them treatment priority. Second, the requirement of a nucleic acid certificate for everyone to enter the hospital may delay the visit of infants to the doctor. As a special group, infants and young children should be exempted from nucleic acid certification. This policy is dehumanizing, in that, first, logistics and city deliveries are required to stop during ECP, which makes it impossible to purchase and deliver infant supplies. This study shows that during the quarantine period, measures that both restrict travel and mandate the suspension of logistics and deliveries have caused a shortage of supplies, as well as the contradiction that the supplies cannot be delivered even if they are available so that the logistics and deliveries should not be suspended at any time to enable the supply needs of infants. Moreover, it is necessary that the supply of public goods should be given priority to infants. Second, the unexpected and random conditions of centralized quarantine lacked consideration of the environment in which the infants were accommodated. This study recommends that families with infants can adopt home isolation measures to avoid the separation of mother and child. It is necessary to note that with the severe global economic decline during the COVID-19 pandemic, the cost of living is increasing invisibly, so that the cost of parenting becomes high, which aggravates the financial burden. The government should transition the strict ECP to a normalized policy to gradually reduce the impact of the pandemic on living conditions (52).

In addition, there were fewer cases of positive emotions obtained in the study, and through interviews, it is assumed that this is because women in the postpartum period are more prone to depression due to greater mood swings as they deal with both physical recovery and infant rearing (53, 54). In addition to the COVID-19 outbreak, the suddenness was completely unexpected for new mothers and caused a disruption in their parenting plans. Furthermore, when a mother is infected with COVID-19 during maternal nurturing, it becomes difficult to cope with the crying infants, especially in the late time or early morning. If an infant is infected, he or she may have a fever and a sore throat, especially since most infants under the age of 1 can only express pain by crying, which adds to the psychological burden on the mothers (55). At present, there have been studies that have found COVID-19 can trigger or exacerbate postnatal depression (56, 57). However, most Chinese citizens are unaware of postnatal depression and do not usually seek psychological help in advance. Therefore, there may already be potential patients of postnatal depression among the interviewees, who are not aware of it. Probably because of personal privacy, they hid their illness. Hence, the government should promote healthcare systems such as family doctors to provide one-on-one support and guidance for the mental health of new mothers.

On the whole, it seems unavoidable to have anxiety and fear of childcare during the pandemic, which is a painful stage. The spread of panic caused by the pandemic should not be underestimated. If psychological adjustment is not carried out in time, new mothers may have a heavy psychological burden before being attacked by COVID-19. Many of our interviewees are first-time mothers. The lack of experience has led to their confusion when making mistakes in raising children. They can only rely on conjecture and online information to find solutions. Furthermore, new mothers need a stable period to gradually adjust and adapt, which requires not only their cooperation but also the concerted efforts of society. Finally, the control and treatment of COVID-19 and the adjustment of the ECP is a public issue. We call on society to give them more attention and support because psychological factors have a significant impact on one's mental and physical health. When people are well-cared, understood, and supported by society, they can improve their physical and mental health, which can help them to eliminate anxiety and depression.

The limitations of this article are as follows: First, it is difficult for this study to record the parenting experience and feelings of all new mothers in China during the COVID-19 pandemic. Since there might be significant differences among some cities, it is impossible to generalize the research results. In future research, it is advised to expand the research sample and use more research methods, such as quantitative methods and mixed research methods, to probe deeper into the mental health problems of new mothers. Second, fewer positive emotions were obtained, and the investigation of positive emotions may lead to new findings, as the attitude of those who are optimistic seems to be crucial when most people have negative emotions due to the COVID-19 pandemic. It will be necessary for future studies to collect more samples, explore the reasons why optimists are positive, and explore their approaches to the management of emotions.

## 5. Conclusion

As a major public health emergency, COVID-19 has refocused public attention on the health system. From the outbreak of the pandemic to its subsequent management and prevention, the health system is faced with not only a medical problem but also a public issue that affects the social fabric. The anxiety of new mothers during the COVID-19 pandemic revealed shortcomings in the emergency response of the Chinese health system in terms of mothers and infants. For example, the concerns of new mothers about being infected exposed the lack of transparency in the news reporting of the pandemic and the lack of awareness of the mutation, the latest symptoms, the treatment options, and the after-effects of COVID-19, therefore, it could be understandable that they had a panic about the virus. Meanwhile, the concerns of mothers about the conditions of isolation revealed the inadequate supply of public goods. Moreover, the anxiety of new mothers about the normal treatment of sick infants is a reflection of the over-managed healthcare system. On the issue of infant feeding, inefficiencies in the emergency supply chain during an outbreak of a public health event were revealed, resulting in a lack of provision of basic supplies for infants. Nevertheless, there is no policy enacted to alleviate the financial stress of new mothers during the pandemic, for example, to reduce the cost of childbirth and parenting. It has also been found that the pandemic is a proper time for health and safety education (58), which indirectly prompts new mothers to grow in their knowledge of infectious disease risks, vaccines, healthy parenting, and public health. In addition, it is also an opportunity to enhance the emergency management capacity of the health system in order to prevent the recurrence of similar problems for mothers and infants. Finally, we appeal to society to give more attention and incentives to new mothers and expect the government to introduce a policy of assistance. Only by building a healthy social environment, we can increase the willingness to have children and maintain a stable demographic structure.

## Data availability statement

The original contributions presented in the study are included in the article/supplementary material, further inquiries can be directed to the corresponding author.

## Ethics statement

Written informed consent was obtained from the individuals for the publication of any potentially identifiable images or data included in this article. The patients/participants provided their written informed consent to participate in this study.

## Author contributions

DZ: conceptualization, methodology, and writing—original draft preparation. CC: software and writing review and editing. All authors contributed to the article and approved the submitted version.
